# Dermoscopic Criteria, Histopathological Correlates and Genetic Findings of Thin Melanoma on Non-Volar Skin

**DOI:** 10.3390/genes12081288

**Published:** 2021-08-23

**Authors:** Cesare Massone, Rainer Hofman-Wellenhof, Stefano Chiodi, Simona Sola

**Affiliations:** 1 Dermatology Unit, Galliera Hospital, 16128 Genoa, Italy; 2 Department of Dermatology, Medical University of Graz, 8036 Graz, Austria; rainer.hofmann@medunigraz.at; 3 Plastic Surgery, Galliera Hospital, 16128 Genoa, Italy; stefano.chiodi@galliera.it; 4 Surgical Pathology, Galliera Hospital, 16128 Genoa, Italy; simona.sola@galliera.it

**Keywords:** melanoma, dermoscopy, pathology, genetic, markers, clinic-pathologic correlation

## Abstract

Dermoscopy is a non-invasive, in vivo technique that allows the visualization of subsurface skin structures in the epidermis, at the dermoepidermal junction, and in the upper dermis. Dermoscopy brought a new dimension in evaluating melanocytic skin neoplasms (MSN) also representing a link between clinical and pathologic examination of any MSN. However, histopathology remains the gold standard in diagnosing MSN. Dermoscopic–pathologic correlation enhances the level of quality of MSN diagnosis and increases the level of confidence of pathologists. Melanoma is one of the most genetically predisposed among all cancers in humans. The genetic landscape of melanoma has been described in the last years but is still a field in continuous evolution. Melanoma genetic markers play a role not only in melanoma susceptibility, initiation, and progression but also in prognosis and therapeutic decisions. Several studies described the dermoscopic specific criteria and predictors for melanoma and their histopathologic correlates, but only a few studies investigated the correlation among dermoscopy, pathology, and genetic of MSN. The aim of this work is to review the published data about dermoscopic features of melanoma, their histopathological correlates with regards also to genetic alterations. Particularly, this review will focus on low-CSD (cumulative sun damage) melanoma or superficial spreading melanoma, high-CSD melanoma, and nevus-associated melanoma.

## 1. Introduction

Dermoscopy is a non-invasive, in vivo technique that allows the visualization of subsurface skin structures in the epidermis, at the dermoepidermal junction, and in the upper dermis; these structures are usually not visible to the naked eye [1,2,3]. Dermoscopy improves the diagnosis of melanocytic lesions in clinical practice, improves the confidence in the diagnosis of benign pigmented lesions, also reducing the number of unnecessary biopsies [1,2,3]. Several meta-analyses proved that dermoscopy increases the sensitivity for the diagnosis of melanoma without decreasing the specificity, compared with the naked-eye examination [1,2,3].

Dermoscopy presents anyhow few limitations: experience and training in dermoscopy are key factors. Moreover, experts, however, may fail to recognize featureless melanomas or melanomas masquerading as inflammatory or benign lesions, such as spitzoid melanomas, amelanotic melanomas, nodular melanomas, nevoid melanomas, desmoplastic melanomas, or verrucous melanomas [4].

However, dermoscopy brought a new dimension in evaluating melanocytic skin neoplasms (MSN) also representing a link between clinical and pathologic examination of any MSN [5,6,7,8,9]. Histopathology is the gold standard in diagnosing MSN, but although precise histopathological criteria for benign and malignant MSN have been defined, in some cases uncertainty remains; therefore, in the last years, various acronyms to describe and report the “gray zone” have been coined (i.e., SAMPUS: “superficial atypical melanocytic proliferation of uncertain significance” or MELTUMP: “melanocytic tumors of uncertain malignant potential”) [6,10].

Another issue is represented by interobserver reproducibility in the diagnosis of MSN, i.e., among dysplastic nevus with severe dysplasia and melanoma in situ [6,10]. Elder et al. demonstrated that rates of diagnostic reproducibility and accuracy were highest among pathologists with board certification in dermatopathology and in those with five or more years of experience [11].

Ferrara et al. showed that the histopathologic criteria in the diagnosis of MSN can work as such, but histopathologic diagnosis can be improved by the clinical data. In fact, histopathologists can apply their criteria in the absence of any clinical information, however, they feel more confident with their diagnosis by the clinic (dermoscopic)-pathologic correlation [12]. Therefore, clinicopathologic, or better dermoscopic-pathologic correlation, can raise the level of confidence in diagnosing MSN and may improve interobserver reproducibility [12].

Melanoma development is a dynamic process. Studies on the growth rate based on digital dermoscopic follow-up and retrospective analysis showed that at least three different biological types of melanoma exist: very slow-growing (lentigo maligna, LM), slow-growing (superficial spreading melanoma, SSM), and fast-growing (nodular melanoma, NM) [13,14,15,16,17].

The WHO classification of Skin Tumors (2018) recognizes nine pathways to melanoma: (1) Low-CSD (cumulative sun damage) melanoma or superficial spreading melanoma, (2) High-CSD melanoma, (3) Desmoplastic melanoma, (4) Malignant Spitz Tumor (Spitz melanoma), (5) Acral melanoma, (6) Mucosal melanoma, (7) Melanoma arising in congenital nevus, (8) Melanoma arising in blue nevus, and (9) Uveal melanoma. Pathways 1 to 7 regard cutaneous melanoma [18].

The incidence of melanoma is rising worldwide. The estimated age-standardized incidence rates of melanoma in men and women worldwide increased from 2.3 and 2.2/100,000 people, respectively, in 1990 to 3.1 and 2.8/100,000 people in 2008. In 2015, the age-standardized incidence rate was 5 per 100,000 persons per year. In the United States, melanoma is the fifth leading cancer in men and women, with an annual incidence rate in 2019 of 27 per 100,000 among non-Hispanic White Americans, 5 per 100,000 among Hispanic Americans, and 1 per 100,000 in Black Americans and Asian/Pacific Islander Americans. Between the early 1970s and 2000, the estimated incidence of melanoma in Central Europe increased from 3 to 4 cases/100,000 inhabitants per year to 10 to 15 cases/100,000 inhabitants per year. Data from the Queensland Cancer Registry for the period 1995 to 2014 confirmed that the incidence of melanoma in Queensland, Australia, is the highest in the world (72 per 100,000 per year) [19].

Melanoma is one of the most genetically predisposed among all cancers in humans. The genetic landscape of melanoma has been described in the last years but is still a field in a continuous evolution [20,21]. Melanoma genetic markers play a role not only in melanoma susceptibility (familial melanoma, see below), initiation, and progression but also in prognosis and therapy [22,23]. In fact, most cases of melanoma are diagnosed at an early stage, when surgical excision can be curative. For high-risk and metastatic melanoma patients, determination of BRAF status (see below) drives their management. Patients harboring the BRAF mutation may receive first-line targeted therapies (i.e., dabrafenib plus trametinib, vemurafenib plus cobimetinib, and encorafenib plus binimetinib) in both metastatic and adiuvant settings, while immunotherapy is the first line in wild-type patients [22,24,25].

Melanoma is one of the most sensitive tumors to immune modulation. Immune checkpoint inhibitors (ICIs) against cytotoxic T-lymphocyte antigen-4 (CTLA-4) and programmed death-1 (PD-1) have dramatically changed the management of both unresectable and metastatic melanoma as well as those at high risk for recurrence after resection [22,24,25].

The determination of BRAF V600 status is mandatory in patients with resectable or unresectable stage III or IV melanoma (Level of evidence 1, grade of recommendation A). The determination of C-KIT and NRAS status is recommended in BRAF wild-type patients (Level of evidence 2, grade of recommendation C). Clinical implications of melanoma subtype with NF1 mutation are still unknown. Radiation therapy may have a palliative role for symptomatic localized areas of disease. Radiation therapy, especially stereotactic radiosurgery, may be particularly important for patients with brain metastases [22,24,25].

While several studies described the dermoscopic specific criteria and predictors for melanoma and their histopathologic correlates, only a few studies have investigated the correlation between dermoscopy and the genetics of MSN.

The aim of this work is to review the published data regarding dermoscopic features of melanoma, and their histopathological correlates with regards also to genetic alterations. Particularly, this review will focus on low-CSD (cumulative sun damage) melanoma or superficial spreading melanoma, high-CSD melanoma, and nevus-associated melanoma.

## 2. Clinico-Pathologic-Genetic Correlation of Melanocytic Skin Neoplasms

Dermoscopy works in parallel to the skin surface, therefore, it analyzes structures on a horizontal plane, while pathology analyzes sections on a vertical plane. The color of the dermoscopic features depends on the histopathological levels of the correlates within the epidermis and the superficial dermis. Pigmented structures are black in the cornified layer, brown at the dermoepidermal junction (DEJ), and gray-blue in the papillary dermis.

Over the last two decades, several scientific studies described the histopathological correlates of most of the dermoscopic criteria of MSN [6,7,26]. Few studies have also correlated to some dermoscopic features with genetic findings.

### 2.1. Globules (Clods)

#### 2.1.1. Dermoscopy

Globules (clods) are defined as round to oval, at times polygonal, brown to brown-gray, or skin-colored structures that may vary in size and arrangement. *Regular globules* are defined as globules that are uniform in size, shape, and color and that are symmetrically arranged throughout the lesion. Thereby, their arrangement can be uniform (also called aggregated brown globules), peripheral (also called peripheral rim of small brown globules), or central. If located at the periphery or in the center, the globules appear together with other criteria of a melanocytic skin tumor. Regular globules are in favor of a benign nevus. *Irregular globules* are defined as globules that vary in size, shape, and color. They do not follow any specific arrangement. Irregular globules can occur in Spitz nevus and melanoma. Peripheral globules reveal a horizontal growing phase [7,26,27,28,29].

#### 2.1.2. Histopathological Correlate

Globules correlate mainly to nests of melanocytes at the DEJ or in the papillary dermis (here also given by melanophages) and correspond to globular or compound nevi. In some cases, globules may also correspond to aggregations of pigmented melanocytes, or even clumps of melanin within the epidermis and in the stratum corneum.

#### 2.1.3. Genetic Findings

Globular nevi and peripheral rim of globules nevi with either predominantly dermal growth pattern or large junctional nests are at least three times more likely to express BRAF V600E than reticular naevi [30,31]. Single nucleotide polymorphisms (IRF4, TERT) have been also reported in globular nevi [30]. The total mutation per megabase is higher in globular than in reticular nevi and nevi with a peripheral rim of globules [32]. UV radiation-induced point mutations and defective DNA repair have been found in globular, reticular nevi, and nevi with a peripheral rim of globules [32]. Irregularly pigmented globules located eccentrically at the periphery of a raised, otherwise homogeneous melanocytic lesion may raise suspicion for a BAP1-inactivated melanocytic tumor (also known as Wiesner nevus or “bapoma”) [33].

### 2.2. Pigment Network (Reticular Pattern)

#### 2.2.1. Dermoscopy

The pigment network is defined as brown or brown-black lines forming a grid of round to rhomboidal meshes over a light brown background. The pigment network is also commonly referred to as a reticular pattern. Depending on the thickness of the network lines and their distribution, two main types of pigment networks can be differentiated. A *typical (regular) pigment network*: The lines of the network are uniform in color and thickness. They are usually thinner than the network holes and typically fade out at the periphery. The typical network is indicative of a benign nevus. An *atypical pigment network* is defined by network lines, which vary in thickness, color, and appear in an asymmetric distribution. The network lines are often thicker than the network holes and may end abruptly at the periphery. Quite often, the atypical pigment network occurs together with white and gray areas. An atypical network is suggestive of melanoma but may be occasionally seen in Spitz nevus [26,27,28,29,30,34,35]. A *negative (inverse) network*, found predominantly in melanoma or Spitz/Reed nevus, consists of serpiginous lighter grid lines that connect between hyperpigmented, elongated-to-curvilinear globules [6,7,26].

#### 2.2.2. Histopathological Correlate

The pigment network corresponds histopathologically to elongated and hyperpigmented rete ridges and the meshes of the network correlate to the dermal papillae and is found in predominantly junctional nevi. The appearance of the pigment network is thus determined by the size and configuration of rete ridges. The irregular pigment network correlates with elongated, enlarged, and hyperpigmented rete ridges with melanin or atypical melanocytes in single units or irregular nests mostly arranged at the DEJ or with pagetoid spread. A negative (inverse) network probably correlates to hypopigmented elongated rete ridges bridging and surrounding large nests of melanocytes within widened dermal papillae [6,7,26,27,28,29,30,34,35].

#### 2.2.3. Genetic Findings

Melanocytic nevi with reticular patterns usually do not express the BRAF V600E mutation [31]. However, Tan et al. found that reticular nevi were 67% BRAF V600E/K and 33% NRAS mutant [36]. Reticular patterned nevi have been associated with single nucleotide polymorphisms (CDKN1B, MTAP, PARP1) [30]. Non-carriers of MC1R R variants develop SSM dermoscopically characterized by an atypical pigment network which is more frequent in darky-pigmented patients and less frequent in individuals with red hair colour [37].

### 2.3. Pseudonetwork

#### 2.3.1. Dermoscopy

Facial melanocytic, but also non-melanocytic skin tumors, are characterized by a pseudonetwork pattern. This pattern is characterized by a diffuse pigmentation, which appears interrupted by multiple round, non-pigmented follicular openings, sweat, and sebaceous glands [6,7,26].

#### 2.3.2. Histopathological Correlate of Pseduonetwork

A pseudonetwork is due to the particular anatomy of the facial skin that is devoid of rete ridges and is characterized by closely situated follicular infundibula and correlates with either melanocytes or pigmented keratinocytes at the DEJ or melanocytes with or without melanophages in the papillary dermis [6,7,26].

### 2.4. Streaks (Pseudopods)

#### 2.4.1. Dermoscopy

Streaks, also called pseudopods, are heavily pigmented, black-gray, or brown peripheral fingerlike projections, at times with a bulbous end. *Regular streaks* (starburst pattern) are symmetrically arranged at the edge of the lesion and arise from a heavily pigmented center. Regular streaks can be seen in Spitz nevus, Reed nevus, and melanoma. *Irregular streaks* are asymmetrically arranged and therefore seen only at some edges of a lesion. Branched streaks are pigmented lines that appear as peripheral prolongations of network lines, whereby the central network often appears over a heavily pigmented background [6,7,26].

#### 2.4.2. Histopathological Correlate

Streaks correlate with peripheral, confluent, and heavily pigmented junctional (or in the papillary dermis) nests of melanocytes independently of the cytomorphologic characteristics of the melanocytes within these nests. The longish, linear shape of the streaks gives evidence to the fact that these pigmented junctional nests of melanocytes form tubules parallel to the skin surface [6,7,26].

#### 2.4.3. Genetic Findings

An HRAS mutation is frequent in Spitz nevi with a typical starburst pattern [32]. Streaks are frequently observed in BRAF mutated melanomas and are considered a sign of tumor growth and proliferation [38,39].

### 2.5. Structureless Pattern

#### 2.5.1. Dermoscopy

A structureless pattern is characterized by a diffuse pigmentation in the absence of any recognizable morphologic pigmented or vascular structures. In melanocytic skin tumors, the structureless pattern may appear monochromatic (blue, brown, gray, black), bichromatic (blue-white, blue-brown, blue-gray, blue-black, brown-gray, brown-black, brown-white), or polychromatic (more than two colors including blue, gray, brown, white, black, and red) [6,7,26].

#### 2.5.2. Histopathological Correlate

The histopathological correlates always reside in the dermis. The blue hue of a common blue nevus correlates with the collection of dentritic melanocytes among the collagen fibers in the reticular dermis. Conversely, deeper melanocytes result in a more blue hue. The structureless monochromatic brown or gray pigmentation of a congenital nevus (intradermal nevus) correlates with a dermal collection or sheets of monomorphic melanocytes in the papillary and reticular dermis, without nests and mainly in sheets, with no junctional component. A structureless bichromatic black and gray pattern may correlate with sheets of melanocytes, melanophages, dentritic, or epithelioid melanocytes. The more superficial are the melanocytes, the browner is the color [6,7,26].

#### 2.5.3. Genetic Findings

Intradermal congenital nevi may express the BRAFV600E mutation [40]. GNAQ is associated with blue nevi [31]. A structureless pattern with an eccentric dots/globules pattern and network pattern with raised structureless areas pattern was identified in BRCA1-associated protein 1 (BAP1)-inactivated melanocytic tumors and were more common in patients with BAP1 germline mutations [33].

### 2.6. Black-Brown-Gray Dots

#### 2.6.1. Dermoscopy

Black-brown-gray dots are loosely arranged tiny dots of black, brown, or gray color.

#### 2.6.2. Histopathological Correlate

Black-brown-gray dots correlate to focal collections (small nests) of melanocytes or clumps of melanin at various levels of the epidermis (mainly in the stratum corneum), at the dermoepidermal junction, and/or in the papillary dermis. The size and shape of dots reflect the extent and form of the accumulation of these pigmented structures, whereas their color depends on the level of these pigmented aggregates within the epidermis and the superficial dermis [6,7,26].

#### 2.6.3. Genetic Findings

Eccentric dots/globules together with a structureless pink-to-tan structureless pattern are common in patients with BAP1 germline mutations [33]. Carriers of the R MC1R variants show dots and globules in nevi [37].

### 2.7. Black Blotches (Structureless Black Pattern)

#### 2.7.1. Dermoscopy

Black blotches (structureless black pattern, hyperpigmented structureless areas) is a diffuse black pigmentation that usually does not occupy the entire lesion. In nevi, the structureless black pattern is usually located in the center. In melanoma, the structureless black pattern does not follow any specific arrangement and appears randomly distributed throughout the lesion [6,7,26].

#### 2.7.2. Histopathological Correlate

Histopathologically, melanin at all levels of the epidermis (but predominantly in stratum corneum) and/or a large melanin-containing dermal area produce the structureless black pattern. In addition, black may also occur in thick melanomas that show pigmented sheets of melanocytes just beneath a very thinned epidermis [6,7,26].

#### 2.7.3. Genetic Findings

Structureless areas have been reported in CDKN2A mutants with two red hair colour-melanocortin 1 receptor (RHC-MC1R) variants. RHC MC1R variant carriers develop melanomas with less blotches of pigment [41].

### 2.8. White Structureless Areas

#### 2.8.1. Dermoscopy

White structureless areas are a diffuse white pigmentation that does not occupy the entire lesion and usually corresponds to a clinically flat part of the lesion [6,7,9,26].

#### 2.8.2. Histopathological Correlate

White depigmentation is caused by fibrosis due to a scar that histopathologically corresponds to thickened fibrotic collagen fibers in the papillary dermis [6,7,26].

#### 2.8.3. Genetic Findings

Structureless areas have been observed in CDKN2A mutants with two red hair colour-melanocortin 1 receptor (RHC-MC1R) variants.

### 2.9. Regression Pattern

#### 2.9.1. Dermoscopy

The regression pattern consists of a combination of diffuse structureless white areas and more or less densely aggregated gray dots (also called gray pepper-like granules or blue-gray peppering). Regression may appear in nevi and melanoma [6,7,9,26].

#### 2.9.2. Histopathological Correlate

The regression pattern is due to extended areas of fibrosis with thickened collagen fibers in the dermis and/or variable amounts of melanophages. Lymphocytes may be present. White areas correspond to fibrosis and blue areas to melanosis (gray pepper-like granules). However, since fibrosis and melanosis are commonly found together, combinations of white and blue areas are often noted also dermoscopically [6,7,9,26].

#### 2.9.3. Genetic Findings

Both structureless white areas and peppering have been described more frequently in BRAF and NRAS mutated melanomas than in wild type [38,39]. Genetic variants in MTAP are associated with regression structures (peppering, mixed regression) [38].

### 2.10. Blue-White Veil (Structureless Blue-White Pigmentation)

#### 2.10.1. Dermoscopy

Blue-white veil (structureless blue-white pigmentation) refers to structureless areas of confluent blue pigmentation with an overlying white “ground-glass” film; thus, the blue-white veil is a variation of the structureless pattern, but cannot occupy the entire lesion and usually corresponds to a clinically elevated part of the lesion [6,7,9,26].

#### 2.10.2. Histopathological Correlate

Histopathologically, the blue-white veil corresponds to an acanthotic epidermis with compact orthokeratosis and more or less pronounced hypergranulosis above sheets of heavily pigmented atypical melanocytes and/or melanophages in the superficial dermis [6,7,9,26].

#### 2.10.3. Genetic Findings

The blue-white veil is also a dermoscopic feature of BRAF and NRAS mutated melanomas and carriers of genetic variants in MTAP, as reported in different studies [38,39,42].

### 2.11. Shiny White Structures (Shiny White Streaks)

#### 2.11.1. Dermoscopy

Shiny white structures (shiny white streaks) is a relatively new criterion, which is only seen using a polarizing light source and consists of relatively short, thick crossing lines. *Reticular depigmentation* consists of white fines crossing lines resembling a white network pattern (negative pigment network, see above) [6,7,9,26,27,28,29,30,34].

#### 2.11.2. Histopathological Correlate

The exact histopathological correlate has not been univocally found yet; the correlation has been proposed with undulation/superficial invagination of the epidermis, compact orto-hyperkeratosis, and alteration of the superficial dermis [6,7,9,26,27,28,29,30,34].

#### 2.11.3. Genetic Findings

Genetic variants in MTAP, PAX3, and IRF4 have been recently associated with shiny white streaks [38].

### 2.12. Vascular Structures

#### Dermoscopy

Vascular structures [red dots (former dotted vessels) and clods (former milky red globules) and linear vessels] correspond to increased vessels (neoangiogenesis) and/or dilated regular/irregular vessels in the superficial dermis [43]. Dotted vessels are more common in wild-type thin melanomas [38].

## 3. Low-CSD Melanoma or Superficial Spreading Melanoma

Low-CSD (cumulative sun damage) melanoma or superficial spreading melanoma (SSM) is the most common subtype, comprising approximately 75% of all malignant melanomas. SSM can arise de novo (DNM: de novo melanoma) or on a pre-existing nevus in about 30% of cases (nevus associate melanoma, NAM) [18].

### 3.1. Clinical Features

**Figure 1:**
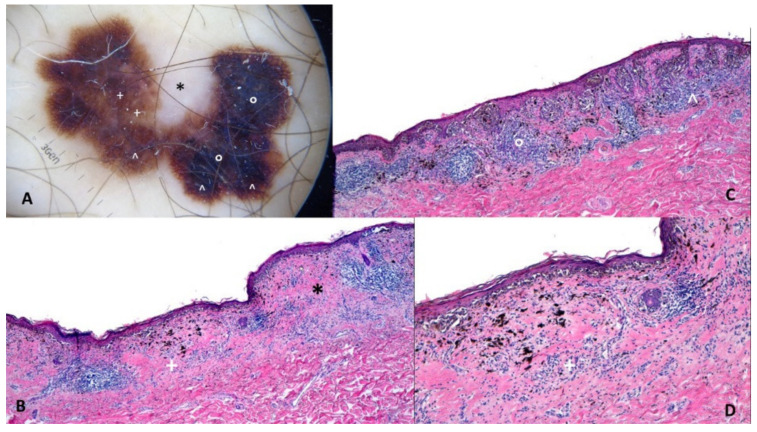
Superficial spreading melanoma (T1a, Breslow 0.5 mm) on the abdomen of an 82-y old man. This de novo slow-growing melanoma shows on polarized contact dermoscopy (Dermalite ProHR^®^, 3Gen, San Juan Capistrano, CA USA) an asymmetric polychromatic multicomponent pattern with atypical pigment network (Panel **A**: labeled ^), white regression (Panel **A**: labeled *), structureless blue pigmentation (Panel **A**: labeled °) and structureless brown-blue bichromatic areas (Panel **A**: labeled +). The atypical network correlates with the hyperpigmented rete ridges with atypical melanocytes in single units and irregular nests mostly arranged at the DEJ (Panel **C**: labeled ^); the presence of a heavy lymphohistiocytic infiltrate with melanophages and acanthosis of the epidermis and heavily pigmented atypical nest and melanocytes in the epidermis are responsible for the structureless blue pigmentation (Panel **C**: labeled °). Panel **B** shows in the dermis fibrosis with a lymphohistiocytic infiltrate that correlates with the area of regression (Panel **B**: labeled *) while the atypical melanocytes in single units and irregular nests mostly arranged at the DEJ and the melanophages in the dermis correlate with structureless bichromatic brown-blue areas (Panel **B**,**D**: labeled +). (original magnification: Panel **B**,**C**: HE, 40×; Panel **D**: HE, 100×).

**Figure 2:**
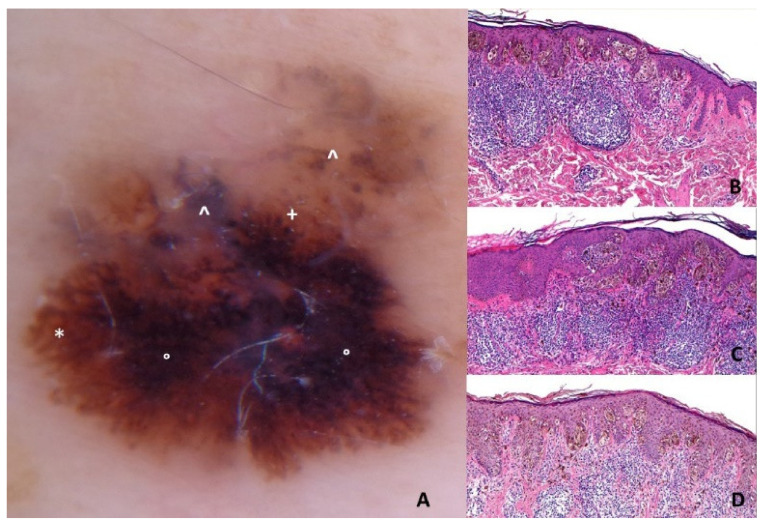
Superficial spreading melanoma (T1a, Breslow 0.53 mm) on the left chest of a 44-y old man. Panel **A**: Polarized contact dermoscopy (Dermaview^®^, Tre T Medical, Camposano, NA, Italy) shows asymmetry, multiple colors, pseudopods (labeled *), atypical network, structureless black areas (labeled °), structureless blue areas (labeled ^), irregular globules (labeled +) and dots. Pseudopods correlate with peripheral, confluent, and heavily pigmented atypical junctional nests of melanocytes (Panel **B**; HE: 100×). Irregular globules correspond to irregularly large atypical nests of melanocytes at the DEJ (Panel **C**; HE: 100×) while irregular dots to small atypical nests of melanocytes or irregular clumps of melanin in the epidermis (Panel **D**; HE: 100×). The structureless black and blue areas are related to the heavy band-like lymphohistiocytic infiltrate with melanophages in the dermis.

Clinically, SSM manifests as a variably pigmented macule or plaque with irregular borders, ranging in size from a few millimeters to several centimeters, often with multiple shades of colors from brown to black (Figure 1 and Figure 2). Biologically, SSM is a slow-growing melanoma over months or years [13]. Therefore, SSM is characterized by an initial radial growth phase (RGP) followed by a vertical growth phase (VGP) with invasion in the dermis. Regression (white areas) and inflammation may be present at any stage. Occasionally, SSM may be also amelanotic. The appearance of a papule or a nodule (eventually also bleeding) is a sign of VGP. Low-CSD melanomas occur mostly on the trunk of younger patients (usually also with multiple nevi) than high-CSD melanomas [18].

### 3.2. Genetic

Genomic mutations and/or aberrations are present in the majority of melanomas (Table 1). It has been demonstrated that low-CSD melanomas mainly (70%) do not originate from preexisting nevi [18,44]. According to the stem-cell cancer model proposed for melanoma, SSM should derive from melanoma stem cells of the epidermal basal layer [14]. Pampena et al. hypothesized that both low-CSD and high-CSD melanomas originate mostly from mutated melanocytes with non-visible precursors while a stepwise pattern within common or atypical/dysplastic melanocytic nevi as precursor occur only in a minority of both low-CSD and high-CSD melanomas [44].

Activations of the mitogen-activated protein kinase (MAPK) pathway by BRAF and NRAS mutations are more common in low-CSD melanoma (60% and 20%, respectively). BRAF oncogene on chromosome 7q34 encodes a serine/threonine kinase; NRAS oncogene is situated on chromosome 1p13.2 and encodes a GTP-binding protein located upstream of BRAF within the MAPK pathway. BRAF and NRAS mutations are mutually exclusive. BRAF V600E mutations are more common in females and younger patients. The second most common BRAF mutation (V600K) is found in patients with an older age of onset and an increased risk for brain and lung metastases [18,45,46].

The TERT (Telomerase Reverse Transcriptase) gene on chromosome 5p15.33 encodes the reverse transcriptase component and the core catalytic subunit of telomerase. TERT promoter mutations that cause increased expression of telomerase (resulting in cell immortalization) are found in most low-CSD melanomas and have been identified as the second earliest alteration required for melanoma development. However, TERT promoter mutations have been also found in nevi with atypia.

A hallmark of UV radiation-induced point mutations, mostly cytosine to thymidine (C > T) transitions, at dipyrimidine sites, are typically found in about 15 mutations/Mb. The (C > T) transition is a signature (UV-signature) associated with ultraviolet (UV) radiation and modifies the p53 pathway. The point mutation burden increases with tumor progression from nevus to melanoma. TP53 and phosphatase and tensin homolog (PTEN) modifications occur in advanced primary melanomas.

#### Familial Melanoma

It is estimated that 10% of cases of melanomas occur in a familial setting as an autosomal dominant trait with incomplete penetrance [47]. In areas of moderate to high incidences of melanoma, families with three relatives with melanoma, individuals with three or more primary melanomas, or individuals with melanoma diagnosed at an earlier age are candidates for hereditary melanoma susceptibility testing [47]. Twenty to sixty percent of these families harbor a mutation in cyclin-dependent kinase inhibitor 2A (CDKN2A) that is the major susceptibility gene. The CDKN2A mutation can also be found in around 10% of sporadic patients with multiple primary melanomas [48,49]. CDKN2A encodes for the cyclin-dependent kinase inhibitor p16 and the nuclear protein p14ARF that regulate the cell cycle at the G1/S checkpoint. Inactivation of CDKN2A occurs by homozygous deletion or sporadic somatic mutation. Biallelic loss of CDKN2A has been proven to be an early event in melanomagenesis and is found in invasive melanomas. The presence of the CDKN2A melanoma susceptibility has been observed also in patients with multiple primary melanoma (MPM) [50].

Less common melanoma susceptibly genes include CDKN2A/ARF, CDK4 (cyclin-dependent kinase 4), TERT, MITF (melanocyte inducing transcription factor), BAP1 (BRCA1 associated protein-1), and POT1 (protection of telomeres 1). Other genes that may soon be included in hereditary melanoma testing include ACD (adrenocortical dysplasia protein), MC1R, RB1 (retinoblastoma susceptibility gene), and TERF2IP (telomeric repeat-binding factor 2-interacting protein 1) [47].

MC1R is the gene responsible for much of the diversity in human pigmentation; two variants have been identified (R and r alleles). The polymorphisms of MC1R are clinically associated with red hair colour (RHC), fair skin, poor tanning ability, and a higher risk for the development of CM. The loss-of-function MC1R variants (“R”) have been strongly associated with red hair color phenotype and increased melanoma risk. Different studies have shown that the presence of at least one MC1R variant is associated with a reduction in age at cutaneous melanoma diagnosis in families with CDKN2A mutations [51]. Medium-risk RHC MC1R variants can increase the genetic risk in CDKN2A mutation carriers by up to 14 times. MC1R r variants are more prevalent in childhood and adolescent melanoma than in adult melanoma [52].

### 3.3. Histopathology

The diagnosis of SSM is based upon a combination of architectural and cytologic criteria. The presence of atypical melanocytes in all layers of the epidermis (pagetoid spread) and architectural disorder (i.e., asymmetry, poor circumscription, nests of melanocytes of various sizes and shapes in the lower epidermis and dermis, absence of maturation) are required for the diagnosis [53]. Solar elastosis with different degrees is constantly observed in the dermis. Tumor invasion is measured by the Breslow index [18]. Neoplastic melanocytes stain positively for S100, Melan-A, and HMB-45 (with loss of gradients as seen in melanocytic nevi). BRAF expression can be assessed also by positive immunostaining by VE1 monoclonal antibody. The immunostain for BAP1 can document the loss of the constitutive nuclear immunoreactivity in BAP1-inactivated melanocytic tumors [18]. A positive anti-PRAME immunostain can favour the diagnosis of a “traditional” melanoma from a nevus as well as loss of p16 by negative staining. Ki67 highlights mitotic figures [54].

#### Tumor Microenvironment

In the last years appeared the clear influence of the tumor microenvironment (TM) and fibroblast population plasticity on melanoma growth, therapy resistance, and immuno-escape. The TM includes normal fibroblasts and melanoma-associated fibroblasts (MAFs) that are flexible cell types interacting with melanoma and stromal cells. In the melanoma setting, normal fibroblasts are forced by tumor cells to acquire a constitutively activated state and differentiate into the tumor-promoting MAFs. The shift from the normal microenvironment to a TM and from normal fibroblasts to MAFs deeply sustains melanoma growth, progression, targeted therapy resistance, and immunosurveillance. In fact, MAFs secrete soluble mediators (such as pro-inflammatory proteins, MMPs, IL-6, HGF, etc.) leading to melanoma growth and progression. MAF-derived molecules, including growth factors, cytokines, chemokines, and proteases promote an extracellular matrix (ECM) remodeling. ECM modified by MAFs becomes a functional and structural support network, and ECM proteins produced by MAFs induce pro-survival, pro-proliferative, and pro-migratory signalling pathways on melanoma cells enabling melanoma cells to proliferate and survive also during pharmacological treatment. Moreover, MAFs generate an immunosuppressed melanoma TM by multiple mechanisms, such as increasing the expression of various inflammatory and immunosuppressive factors; among others, TGF-ß, IL-6, MMPs, PGE2, COX-2, CXCL5, and PDL1/2, which dramatically impair the anti-tumor activity of immune cells. Furthermore, MAFs alter the extracellular availability of lactate, glucose, and arginine, with the final generation of an immunosuppressive, glucose- and arginine-poor, lactate-rich TM which allows melanoma cells to evade immune surveillance and thus survive and proliferate safely in the tumor mass. Among ECM proteins, matrix metalloproteinases (MMPs) are a class of peptidase able to remodel the ECM by favoring the tumor invasive processes. Particularly, MMP-9 is the most involved in melanoma, being implicated in neoangiogenesis, ECM degradation, tumor invasion, cell migration, and formation of metastasis. Dysregulation of the MAPKs and PI3K/Akt signaling pathways, TGF-ß pathway, and osteopontin can lead to an aberrant overexpression of MMP-9. Moreover, epigenetic events may alter the expression of MMP-9. In fact, different studies have identified specific ncRNAs associated with melanoma or miRNAs able to selectively target and modulate MMP-9. Interestingly, several studies have proposed MMP-9 as a candidate marker used for the assessment of dabrafenib therapeutic response. Salemi et al. proposed MMP-9 as a new biomarker of response or resistance to the treatment. Moreover, the development of new selective inhibitors for MMPs (MMPs inhibitors) could represent a new promising strategy in melanoma therapy [23,55,56,57].

### 3.4. Correlation between Dermoscopic Criteria, Histopathology and Genetic Findings

#### 3.4.1. Early Superficial Spreading Melanoma

The radial phase of growth of SSM is characterized by the pagetoid spread of atypical melanocytes in all layers of the epidermis. This is an architectural and cytological dynamic process. Typically, initially, the intraepidermal component extends laterally for more than three rete ridges past the confines of the dermal component [38]. Neoplastic melanocytes became progressively larger and often of epithelioid morphology and are arranged in either single units or atypical nests [2]. Viros et al. found that an increased upward scatter and nest formation of intraepidermal melanocytes in the RGP portion of the tumors, thickening of the involved epidermis, sharp demarcation from the surrounding skin, as well as the presence of a larger, rounder, and more pigmented tumor cells were distinguishing features of melanomas with BRAF mutation and tend to occur in the skin without marked solar elastosis in anatomic sites such as the trunk. A proposed binary decision tree was able to predict a BRAF mutation status with 60.3% accuracy [58]. Broekaert et al. tested alternative trees and confirmed the previous findings of Viros et al. [59].

Unfortunately, extensive and exact studies on dermoscopic-pathologic-genetic correlation in SSM are still lacking. Therefore, based on the experience of Viros et al., it could be speculated that the initial histopathological features of an early SSM and their dermoscopic correlates are probably more frequent in BRAF mutated SSM than in WT SSM (Table 2). However, BRAF mutations alone cannot be responsible for all dermoscopic-pathologic correlates in SSM because BRAF mutation is also frequently observed in melanocytic nevi.

The atypical pigment network reveals disarrangement of the rete ridges with pleomorphic melanocytes and changes in melanin distribution in the epidermis [6,7,26]. Shi et al. recently confirmed with univariable and multivariable analysis that thick brown lines correlate with pigmented epidermis [60]. An atypical network frequently (88%) causes the presence of eccentric hyperpigmentation on dermoscopy [61,62]. The lentiginous melanocytic proliferation along with the DEJ characteristic of the lentiginous melanoma on sun-damaged skin of the elderly also correlates with an atypical network on dermoscopy [63,64]. Non-carriers of MC1R (Melanocortin-1 Receptor) R variants develop SSM dermoscopically characterized by an atypical pigment network which is more frequent in darky-pigmented patients and less frequent in individuals with red hair colour [37].

At this stage, small irregular nests of atypical melanocytes and collections of melanin correspond with the irregular dots observed on dermoscopy and their size and shape correlate with the extent and form of the accumulation of these pigmented structures. The color depends on their level within the epidermis and the superficial dermis [6,7,26].

When larger pigmented junctional (or in the papillary dermis) irregular nests of atypical melanocytes form tubules parallel to the skin surface at the periphery of the lesion, irregular streaks became visible on dermoscopy. Streaks are frequently observed in BRAF mutated melanomas [38,39].

Large irregular nests of atypical melanocytes at the DEJ or in the superficial dermis corresponds to irregular globules on dermoscopy [6,7,26].

Areas of heavily pigmented melanin at all levels of the epidermis (but predominantly in stratum corneum) and/or in the superficial dermis may develop and appear on dermoscopy as either multiple, small, irregularly shaped, and bizarrely outlined dark areas (so-called irregular hyperpigmented areas), or relatively large, roundish, hyperpigmented, structureless zone (black blotches), [65] often observed eccentric in melanoma (dermoscopic island) [62].

Regression is frequently observed, particularly in melanoma in situ. Histologically, early regression features show moderate to dense lichenoid infiltrate in the upper dermis; in fully developed regression, lymphocytes have been replaced by melanophages and fibrosis. Early regression may be rarely seen on dermoscopy as an asymmetric irregular homogeneous pigmentation with asymmetric vitiligoid halo (so-called “halo nevus-like melanoma”) [66]. Granularity/peppering (dots, blue-gray) and scar-like depigmentation (structureless zone, white) are the dermoscopic features of fully developed histological regression [6,7,9,26]. Peppering is frequently found in BRAF mutated melanomas [38,39]. The presence of mixed regression with white and blue areas and the presence of regression in more than 50% of the lesion are strongly associated with melanoma, being more associated with invasive melanoma than in situ melanoma [9].

**Figure 3:**
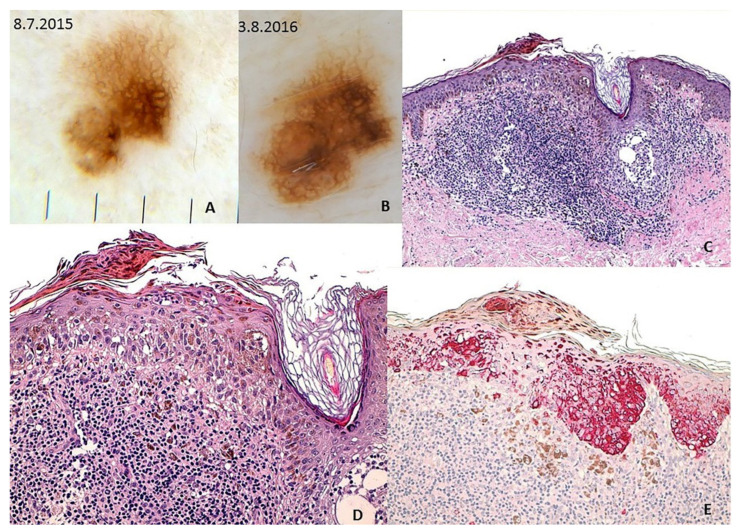
Melanoma in situ on the left flank of a 62-y old man. This small diameter melanoma showed upon polarized contact dermoscopy (Dermaview^®^, Tre T Medical, Camposano, NA, Italy) an asymmetric growth at 1-year digital follow-up (Panel **A**,**B**), atypical network, structureless brown areas, and irregular pigmented areas. Histology (Panel **C**; HE, ×100) shows a proliferation of atypical melanocytes in single units and nest in all layers of the epidermis and a dense lymphohistiocytic infiltrate with melanophages in the dermis. Pagetoid spread is confirmed also by Melan-A staining (Panel **E**, 200×). The irregular pigmented areas correlate with the clumps of melanin and pigmented parakeratosis (Panel **D**; 200×); the structureless brown areas correlate with the dense lymphohistiocytic infiltrate with melanophages in the dermis.

Lallas et al. recently proved that atypical pigment network, irregularly pigmented areas, regression, prominent skin marking, and angulated lines are the five main positive dermoscopic indicators of melanoma in situ [66]. Irregular hyperpigmented areas usually correlate with clumps of melanin or hyperpigmented atypical melanocytes in the epidermis or stratum corneum. Angulated lines are similar to the rhomboidal structures of LM (see below) [66]. Atypical dots/globules and irregular hyperpigmented areas (Figure 3) are also the most prevalent dermatoscopic features in small diameter melanoma (<5 mm) [67].

#### 3.4.2. Invasive Superficial Spreading Melanoma

If SSM became invasive it would have a slower vertical growth rate than NM [15,68]. In the literature, it has been reported that at diagnosis SSM has a median thickness ranging from 0.88 to1.1 mm, in some studies up to 2.26 mm [68].

When the radial growth phase occurs, there is an invasion in the dermis by atypical melanocytes arranged in irregular large nests or sheets of neoplastic cells together with melanophages and the epidermis may become acanthotic with areas of hypergranulosis and orthokeratosis. At this point, a blue whitish veil and shiny white streaks are observed upon dermoscopy. The most important indicator of a BRAF mutation is the presence of the blue-white veil [42]. Genetic variants in MTAP, PAX3, and IRF4 have been recently associated with shiny white streaks [38]. Shit et al. recently confirmed that shiny white streaks and negative networks are the most predictive features of melanoma or severe atypia and both correlate with increased fibroplasia [29,30,34,60]. In addition, atypical vessels, polymorphous vessels, and milky red areas became prominent and ulceration may occur [69]. Blue-whitish veil, atypical pigment network, more than three colors, and milky-red areas, are also related to a >1/mm^2^ mitotic rate, and to the presence of distant metastases [41].

Recently, Sgouros et al. reported that light brown coloration (50.7%) and irregular brown dots/globules (42.0%) were most frequently observed in ≤2 mm NMs. Multivariate analysis revealed that dotted vessels (3.4-fold), shiny white streaks (2.9-fold), and irregular blue structureless area (2.4-fold) were predictors for thinner NM [70].

#### 3.4.3. Amelanotic/Hypomelanotic Melanoma

Amelanotic/hypomelanotic melanoma (AHM) is a subtype of melanoma that may be difficult to diagnose because of a lack of pigmentation and the presence of symmetry. Thin amelanotic/hypomelanotic superficial spreading melanomas (AHSSM) are dermoscopically more difficult to diagnose than pink thick melanomas. Homogeneous, pinkish-red pattern with polymorphic vessels and the combination of dotted and linear irregular vessels are the most relevant dermoscopic criteria for thin AHSSM; milky-red areas occur more frequently in thick AHM [69,71,72].

#### 3.4.4. Familial Melanoma

Early melanomas from CDK2NA mutation carriers tend to develop melanomas with less dermoscopic structures and less colors, or hypopigmented and are more difficult to diagnose even with the comparative approach and benefit from the detection of changes during digital dermoscopy monitoring [48].

Melanomas from non-carriers of MC1R R variants frequently show an atypical pigment network on dermoscopy and are usually SSM, exhibit more dark brown and black colors, asymmetry and structures [41].

MC1R R carriers develop melanomas with reduced dermoscopic structures, lower total dermoscopy score, lower prevalence of atypical pigment network, and less blotch of pigment [37,41]. MC1R RHC variants have been described in hypopigmented melanomas. The presence of an atypical vascular pattern has been also reported in carriers of MC1R R variants [37].

For each patient with multiple primary melanomas harboring a CDKN2A germline mutation, it is possible to find the same kind of dermoscopical pattern among their melanocytic tumors [50].

## 4. Nevus Associated Melanoma

NAMs are placed in the 2018 WHO classification “low to no cumulative solar damage” category [37]. However, NAMs represent only a proportion of low-CSD melanomas (about 30%), as the majority of low-CSD melanomas do not originate from pre-existing nevi [44].

### 4.1. Clinical Features

Clinically, NAMs appear to be an SSM occurring as an irregularly pigmented macule mostly on the trunk and less on the extremities of patients with a history of sunburns (>5) and high nevus count. Depending on the stage of development, further important frequent features are irregular borders, multiple colors, and dimensions > 6 mm. A rapidly growing and even bleeding papule or nodule indicate dermal invasion [18,44,73].

In a recent meta-analysis, Pampena et al. found that NAMs were more frequently associated with intradermal nevi (54.0%) than with junctional (21.7%). The data showed a slight nonsignificant trend for NAMs to be associated more frequently with nondysplastic nevi than dysplastic nevi (RR 0.77, 95% CI 0.49–1.20) [74]. Caccavale et al. found that 77.8% C-NAMs arose on small congenital nevi < 1.5 cm [75].

### 4.2. Histopathology

The histopathological criteria for melanoma arising in a pre-existing nevus are the same as described for SSM; per definition, remnants of the original benign nevus are found in the epidermis or more frequently in the dermis. Cymerman et al. and Pampena et al. found a lower Breslow thickness in NAMs than in DNMs: a possible explanation may be that nevus cells might have obscured malignant cells with difficulties in assessment Breslow measurement [44,73]. Dermoscopic features correlate with the same histopathological counterpart as above.

### 4.3. Genetic

Genetic analysis found that associated nevi were BRAF V600E mutated. BRAF V600E mutations are the most frequent and earliest events that occur on low-CSD melanoma and associated nevi. In fact, BRAF V600E is associated with large junctional nests of acquired nevi or dermal and congenital growth patterns in congenital nevi [76].

Tschandl et al. found BRAFV600 mutations in 63% of melanomas and 65.2% of the nevus component, and NRAS mutations in 10.9% of melanomas and 15.2% of the nevus component [40]. Melanoma arising in giant congenital nevi harbor the NRAS p.Q61 mutation while the BRAF mutation or fusion is infrequent [18].

TERT promoter mutations are also very frequent (77%). Loss of both cyclin-dependent kinase inhibitor 2A (CDKN2A) alleles and the occurrence of genomic copy number alterations are also possible in invasive melanomas [77].

Shain et al. found BRAF V600E in the univocally benign melanocytic proliferation of a NAM with the absence of additional driver mutations while TERT promoter mutation was present in the “intermediate melanocytic proliferation” [78].

### 4.4. Dermoscopic Features

**Figure 4:**
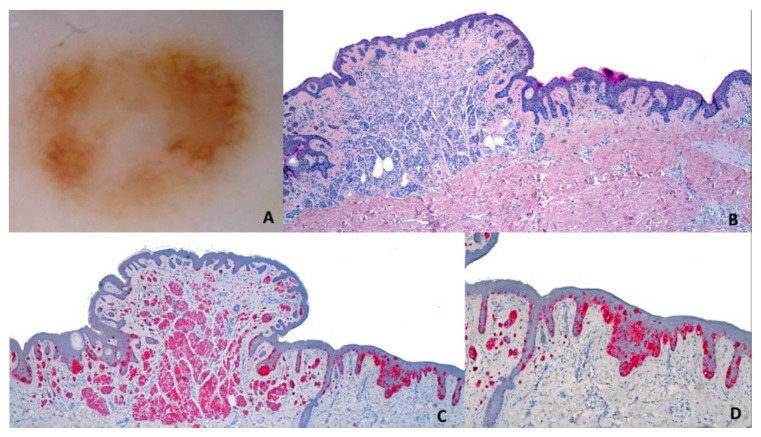
Nevus-associated melanoma in a 76-y old woman on the right buttock. Polarized contact dermoscopy (Panel **A**; Dermalite ProHR^®^, 3Gen, San Juan Capistrano, CA, USA) shows an asymmetric lesion with central hypopigmented-brown structureless areas and an atypical network on the right side. Histology shows an asymmetric melanocytic lesion composed on the left side by a dermal proliferation of monomorphous melanocytes with maturation (corresponding to the central hypopigmented-brown-structureless areas) and on the right shoulder elongated rete-ridges with atypical melanocytes in single units and irregular nests in all the epidermal layers, correlating to the atypical network (Panel **B**; HE: 20×). The Melan-A staining highlights the pagetoid spread (Panel **C**: ×20; Panel **D**: 200×).

Di Stefani et al. reported that in 67.1% of NAM, benign dermoscopic features can still be observed (Figure 4), more frequently a typical pigment network (36.8%), a homogeneous pattern (27.6%), and regular globules (27.6%) [79].

Zalaudek et al. found that melanomas associated with congenital nevi (C-NAMs) occur at a younger age and have a thicker Breslow depth than melanomas associated with acquired nevi (NC-NAMs). Dermoscopically, C-NAMs are more often central to a congenital nevus, with a clod/globular or structureless brown pattern, typical of young patients. The structureless monochromatic brown or gray pigmentation of a congenital nevus correlates with a dermal collection or sheets of monomorphic melanocytes in the papillary and reticular dermis, without nests and mainly in sheets, with no junctional component [73]. The negative network is highly specific especially for NAM, although it can also be seen in Spitz nevi and rarely in congenital nevi [30,34,80].

NC-NAMs are frequently hypopigmented nodules/plaques, eccentric/peripheral, with hypopigmented structureless areas, typical of older patients [73]. The majority of CMN-associated melanomas (63%) exhibited a globular dermoscopic pattern in their benign part, while a blue-white veil and irregular blotches were the most frequent dermoscopic features in the malignant part [75].

Recent studies confirmed that regular peripheral globules are a dermoscopic feature that is mostly associated with benign melanocytic lesions. Melanocytic lesions with atypical or asymmetrically distributed peripheral globules, especially when located on the extremities, should raise suspicion for malignancy [81].

## 5. High-CSD Melanomas/Lentigo Maligna Melanoma

Lentigo maligna (LM) is classified under the “high cumulative solar damage” heading [18]. LM is biologically a very slow-growing melanoma occurring on the chronically sun-exposed skin of the head and neck region. LM is characterized by a long radial growth phase in which it is a melanoma in situ; then, after years, became invasive and confer risk of metastatic disease (Lentigo Maligna Melanoma, LMM) [18,82].

### 5.1. Clinical Features

LM appears mostly on the head and neck area (with the cheeks accounting for 53.7% of the cases), but extra-facial LM/LMM can also be encountered (usually on the trunk in men or extremities in women). LM clinically presents as a slowly growing isolated large pigmented macule or patch with irregular borders. Rarely, LM can also present as an amelanotic/hypomelanotic macule or patch, especially in fair-skinned individuals. With time, LM may evolve into papules, nodules, or thick plaques becoming LMM [18,82].

Frequently, other signs of CSD skin are also present, namely solar elastosis, actinic keratoses, and the number of solar lentigines [18,53].

### 5.2. Histopathology

Therefore, histologically, early LM is characterized mostly by a horizontal proliferation of uniformly atypical melanocytes (with variable nuclear atypia and oft dendritic appearance) and few irregular nests distributed along the DEJ rather than a pagetoid spread, in a thin epidermis (lentiginous pattern) with involvement of hair follicle; the dermis shows constantly solar elastosis, melanophages may be present in variable amount together with a patchy or band-lymphocytic infiltrate [18,53]. A second pattern is characterized by irregular nests of atypical melanocytes with bridging and elongated rete ridges, simulating a dysplastic nevus (dysplastic-nevus-like LM or nevoid LM). LM and LMM are less likely associated with a preexisting nevus than SSM; conversely, LM is found in the overlying epidermis of about 50% of desmoplastic melanomas [18].

### 5.3. Genetic

According to the stem-cell cancer model proposed for melanoma, LM derives from melanoma stem cells of the outer sheet of the hair follicle [14]. High-CSD melanomas carry about 10^5^ somatic mutations per genome (30 mutations/Mb of DNA). The UV signature (pyrimidine dimer formation) is typical [18]. Genetic analysis disclosed that LM and LMM are characterized by loss of NF1 or mutually exclusive activating mutations of the oncogenes NRAS (0 to 15%), BRAF (6 to 22%), and KIT (see Table 2 for details). KIT mutations are more common (15 to 30%) than in low-CSD melanoma (<1%). BRAF mutations are generally not V600E; the BRAF V600K mutation was significantly associated with older age, male sex, head and neck primary melanoma site, a higher degree of chronic sun damage, and short overall survival [45,53].

### 5.4. Correlation between Dermoscopic Criteria, Histopathology

According to the body area, two different settings are recognized.

#### 5.4.1. Melanoma Occurring on Facial Chronically Sun Exposed Skin

**Figure 5:**
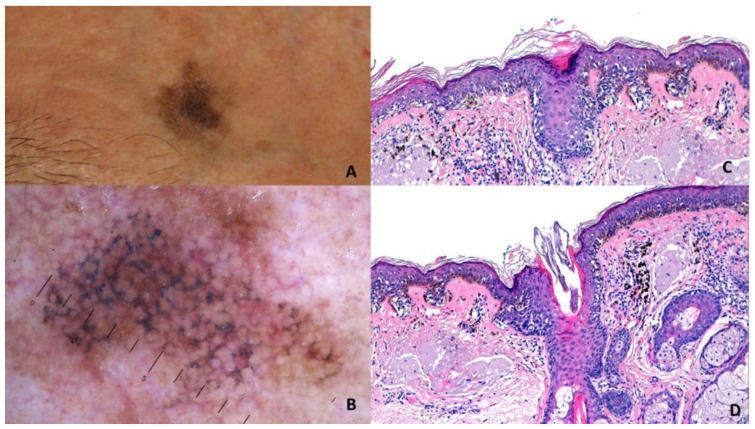
Lentigo maligna on the left temporal region of a 77-y old man. Panel **A**: asymmetric, irregularly pigmented macule. Panel **B**: polarized contact dermoscopy (Dermaview^®^, Tre T Medical, Camposano, NA, Italy) shows asymmetry and multiple colors; asymmetric pigmentation of the hair follicles, dots, and annular-granular pattern. Panel **C**,**D**: Histology shows atypical melanocytes and irregular nests at the DEJ and in the epidermis with involvement of the hair follicles (correlate with the asymmetric pigmentation of the hair follicles); in the dermis, there is prominent solar elastosis, melanophages (correlate with the dots and annular-granular pattern) and a discrete lymphohistiocytic infiltrate. (Panel **C**,**D**: HE, 400×).

LM observed at this stage very well correlate on dermoscopy with asymmetric pigmentation of the follicular openings (gray circles/semicircles), dots around hair follicles with subtle annular-granular pattern and short streaks [83]. The asymmetric pigmentation correlates to atypical melanocytes as single units or small nests in the epidermis that are surrounding and/or extending down hair follicles. Brown dots correspond, on histopathology, to aggregates of melanocytes and small nests at the dermoepidermal junction between the follicles. Blue-gray granularity corresponds to melanophages in the upper dermis [82,84]. With histological progression and an increase in atypical melanocytes not only at the DEJ but with pagetoid spread and formation of atypical nests of melanocytes together with underlying melanophages in the papillary dermis, on dermoscopy became prominent the annular granular pattern and the rhomboidal structures. Progressively, melanin and pigmented atypical melanocytes at all levels of the epidermis (but predominantly in stratum corneum) and/or a large melanin-containing dermal area develop, and homogeneous structureless black areas with obliteration of the hair follicles are visible on dermoscopy (Figure 5). At this stage, invasion in the papillary dermis is usually present. When the vertical growth phase is fully developed, dermoscopical milky-red areas on the nodular component are usually present [26,83].

#### 5.4.2. Melanoma Occurring on Non-Facial Chronically Sun Exposed Skin

Melanoma occurring on chronically sun-exposed skin outside the face presents the same histopathological criteria as described above. Upon dermoscopy, these melanomas may present with three main patterns: angulated lines (correlate to confluent atypical melanocytes along an attenuated DEJ and melanophages in the papillary dermis), patchy peripheral pigmented island (PPPI: a combination of atypical network or structureless areas at the periphery and scar-like or hypopigmented central areas), and tan structureless areas with granularity. The latter two correlate to histopathology with a regression with a variable degree of fibrosis and amount of melanophages in single units or a collection in the dermis, with (in PPPI) at the periphery atypical melanocytes in single units or irregular nests mostly arranged at the DEJ [26,85].

Recently, Peruilh-Bagolini et al. confirmed that gray circles/semicircles and angulated lines are negative predictors of invasive LM while obliterated follicles, irregular blotches, and black color positively predicted invasive LM. These findings are important when incisional biopsies on LM are performed: selecting the area to biopsy with dermoscopy allows to not underestimate the invasive component [86].

## 6. Conclusions

Dermoscopic–pathologic correlation enhances the level of quality of MSN diagnosis and increases the level of confidence of pathologists also by improving the interobserver reproducibility. The gray zone or area of uncertainty on pathology is reduced and more precise diagnoses are given, with a considerable impact on prognosis and the management of patients.

For clinicians, i.e., knowing dermoscopy and pathological correlates, incisional biopsies on LM are performed in more correct areas, avoiding underestimation of the Breslow-index. For pathologists, i.e., information on an asymmetric growth of an MSN at follow-up with digital dermoscopy monitoring is helpful in achieving the diagnosis of early melanoma. For both clinicians and pathologists, i.e., knowing the presence of susceptibility genes in a given patient lifts the level of awareness in both the dermoscopic visit and pathologic evaluation.

While several studies described the dermoscopic specific criteria and predictors for melanoma and their histopathologic correlates, only a few studies investigated the correlation among dermoscopy, pathology, and genetic of MSN. In fact, extensive and exact studies on specific dermoscopic–pathologic–genetic correlations in melanocytic nevi, SSM, DNM, NAM, and LM/LMM are still lacking. Further studies on this issue are needed in order to reduce the gray area of uncertainty in MSN diagnosis allowing better and more appropriate management of our patients.

## Figures and Tables

**Table 1:** Melanomas and genomic mutations and/or aberrations.

Melanoma	Genomic Mutations and/or Aberrations
*Melanoma on intermittently sun exposed skin (Low-CSD melanoma)*	❖ BRAF (*somatic mutation in exon 11 and 15*) 60% of cases. ➢ BRAF V600E (substitution from valine to glutamic acid at codon 600; 74–92% of cases): more common in females and younger patients. Found also in 73–82% of acquired melanocytic nevi ➢ BRAF V600K (substitution from valine to lysine at codon 600): frequent in patients with an older age of onset ➢ BRAF V600R and K601: rare subtypes ❖ NRAS (*somatic mutation*) 20% of cases; found also in congenital melanocytic nevi and some congenital melanocytic nevi ❖ TERT (*promoter mutation*): cysteine to threonine mutations at position 228 (C228T) and 250 (C250T) found in 86% of cutaneous melanomas (BRAFV^600^ or NRAS mutated) but also in atypical nevi ❖ TP53 (*loss-of-function mutation*): occur in advanced primary melanomas ❖ PTEN (*loss-of-function mutation*): occur in advanced primary melanomas ❖ Chromosomal abnormalities: loss of chromosomes 9, 10, 6q, 20; gain of chromosomes 1q, 6p, 7, 8q, 17q, 20q
*Familial melanoma*	❖ CDK2NA (*loss-of-function mutation*): major susceptibility gene but can also be found in around 10% of sporadic patients ❖ Less common melanoma susceptibly genes include CDKN2A/ARF, CDK4, TERT, MITF, BAP1, POT1, ACD, MC1R, RB1, and TERF2IP
*Nevus associated melanoma*	❖ BRAF V600E in about 50% of NAM ❖ NRAS (*somatic mutation*); NRAS p.Q61 in giant congenital nevi ❖ TERT (*promoter mutation*): very frequent (77%) ❖ CDK2NA (*loss-of-function mutation*): occur in invasive melanomas
*Melanoma on chronically sun exposed skin* *(High-CSD melanoma)*	❖ UV-signature (point mutations C > T) ❖ TP53 (*loss-of-function mutation*) ❖ NRAS (*somatic mutation*): most common mutations occur at position 61 where a glutamine is substituted by arginine (NRAS-Q61R), lysine (NRAS-Q61K) or leucine (NRAS-Q61L) ❖ BRAF generally non V600E ❖ NF1 (*loss-of-function mutation*): may occur in BRAF/NRAS double wild-type melanoma patients ❖ KIT (*gain-of-function mutation*): screening in exons 9/11/13 is suggested in BRAF/NRAS double wild-type melanoma patients ❖ CDK2NA (*loss-of-function mutation*): occur in invasive melanomas ❖ TERT (*promoter mutation*): found in melanomas BRAFV^600^ or NRAS mutated ❖ RAC1 (*gain-of-function mutation*): C > T transition with a proline to a serine substitution (UV-signature) frequent in BRAF/NRAS double wild-type melanoma patients

Table legend: C: cytosine. T: thymidine. CSD: cumulative sun damage.

**Table 2:** Dermoscopic criteria, histopathological correlates, and genetic findings of SSM.

Melanoma	Histopathologic Features	Dermoscopic Features	Genomic Mutations and/or Aberrations
Early superficial spreading melanoma	Increased upward scatter and nest formation of intraepidermal melanocytes, thickening of the involved epidermis, sharp demarcation from the surrounding skin, a pigmented tumor cells	Atypical pigment network	BRAF mutation? Non-carriers of MC1R R variants develop SSM dermoscopically characterized by an atypical pigment network
	Small irregular nests of atypical melanocytes and collections of melanin	Irregular dots	BRAF mutation?
	Peripheral, confluent, and heavily pigmented junctional (or in the papillary dermis) nests of melanocytes	Irregular streaks	BRAF mutation
	Large irregular nests of atypical melanocytes at the DEJ or in the superficial dermis	Irregular globules	BRAF mutation?
	Heavily pigmented melanin at all levels of the epidermis (but predominantly in stratum corneum) and/or in the superficial dermis	Irregular hyperpigmented areas	BRAF mutation?
	Areas of fibrosis with thickened collagen fibers in the dermis and/or variable amounts of melanophages	Regression Scar-like depigmentation Granularity/peppering	BRAF mutation NRAS mutation
Invasive superficial spreading melanoma	Acanthotic epidermis with compact orthokeratosis and more or less pronounced hypergranulosis above sheets of heavily pigmented atypical melanocytes and/or melanophages in the superficial dermis	Blue whitish veil	BRAF mutation NRAS mutation Carriers of genetic variants in MTAP
	Undulation/superficial invagination of the epidermis, compact orto-hyperkeratosis, and alteration of the superficial dermis	Shiny white streaks	MTAP, PAX3 and IRF4
	Neoangiogenesis	Atypical vessels, polymorphous vessels, and milky red areas	

Table legend: SSM: superficial spreading melanoma.
